# CDK4/6 inhibitors as conditional immune-priming agents in breast cancer: therapeutic windows, sequencing strategies, and rational combinations with immunotherapy, radiotherapy, and antibody-drug conjugates

**DOI:** 10.3389/fimmu.2026.1934879

**Published:** 2026-08-19

**Authors:** Chenhui Zheng, Yue Pan, Danni Zheng, Yin Pan

**Affiliations:** Department of Surgical Oncology, Taizhou Central Hospital (Taizhou University Hospital), Taizhou, Zhejiang, China

**Keywords:** antibody-drug conjugates, breast cancer, CDK4/6 inhibitors, immune checkpoint inhibitors, immune priming, radiotherapy, therapeutic window, tumor microenvironment

## Abstract

Cyclin-dependent kinase 4 and 6 (CDK4/6) inhibitors are established components of endocrine therapy for hormone receptor-positive (HR+), HER2-negative breast cancer. Beyond RB-dependent G1 arrest, these drugs alter antigen presentation, interferon signaling, checkpoint-ligand expression, suppressive immune-cell populations, and CD8+ T-cell fate. The direction and durability of these effects vary across tumors and treatment settings. Early combinations with immune checkpoint inhibitors (ICIs) have produced activity signals, but hepatotoxicity and pneumonitis have limited several regimens. Radiotherapy and antibody-drug conjugates (ADCs) offer different routes to antigen release and innate immune activation. Therapeutic vaccination and dendritic-cell support provide a further route to exploit treatment-induced antigen display, although this rationale remains preclinical. We examine CDK4/6 inhibitors as conditional immune-priming agents whose value depends on baseline immune contexture, tumor-cell-cycle dependence, endocrine therapy, drug and dose, exposure duration, partner treatment, and organ reserve. Here, immune priming is an operational term for a treatment-induced immune state that a subsequent therapy may use; it does not imply proven *de novo* activation of naïve T cells. Evidence is separated into clinical outcomes, human translational findings, preclinical mechanisms, and testable hypotheses. This synthesis supports a therapeutic-window strategy in which a defined exposure is assessed for a measurable immune change before treatment is escalated. The immediate translational challenge is to identify that window, determine when it can be exploited, and recognize when continued exposure is more likely to reinforce resistance or toxicity.

**Systematic review registration:**

https://clinicaltrials.gov/study/,identifier NCT05766410.

## 1 Introduction

CDK4/6 inhibitors have changed the treatment of HR+, HER2-negative breast cancer. Palbociclib, ribociclib, and abemaciclib improve progression-free survival, and several trials have shown overall-survival benefit when these agents are paired with endocrine therapy in advanced disease. Abemaciclib and ribociclib have also entered the treatment of selected early breast cancers (1–6). Their canonical effect is RB-dependent cell-cycle arrest: inhibition of cyclin D-CDK4/6 preserves RB-mediated repression of E2F transcription and restricts G1-to-S progression in tumors that remain dependent on this pathway (7).

Cell-cycle arrest is only part of the pharmacology. CDK4/6 inhibition can increase tumor antigen presentation and interferon signaling, alter checkpoint-ligand expression, reduce selected suppressive immune populations, and influence T-cell activation and memory (8–15). These effects are not uniformly favorable. Preclinical models often show cooperation with PD-1 or PD-L1 blockade (8–10), whereas human studies describe changes in regulatory T cells (Tregs), myeloid-derived suppressor cells (MDSCs), and activated CD8+ T cells during treatment (15). Clinical combinations have been less consistent. Palbociclib, pembrolizumab, and letrozole showed feasibility and activity in a small phase I/II study, while abemaciclib plus pembrolizumab and ribociclib plus spartalizumab were constrained by toxicity or limited efficacy (16–18). The contrast between mechanistic promise and clinical performance is the central problem addressed in this review.

Throughout this review, conditional immune priming denotes a measurable, time-limited treatment-induced state that improves tumor visibility, relieves suppression, or changes effector-cell state in a way that a second therapy could use. The phrase is operational. It does not assert that CDK4/6 inhibition initiates bona fide *de novo* priming of naïve T cells. A usable state requires a responsive cell-cycle axis, greater tumor visibility, preserved effector function, relief of relevant suppressive circuits, and sufficient safety reserve. When one of these conditions fails, continued exposure can be immunologically neutral, reinforce a resistant niche, or make the combination clinically unusable. The practical question is which patient, drug, schedule, partner, and monitoring strategy can convert immune modulation into therapeutic gain.

Literature search and evidence handling. Structured searches of PubMed, Web of Science, Crossref, and ClinicalTrials.gov were conducted from October 2024 through May 2026 and updated by citation verification and targeted searches in July 2026. Search terms combined CDK4/6 inhibitors with immunity, immunotherapy, radiotherapy, ADCs, endocrine therapy, biomarkers, immunopeptidomics, therapeutic vaccination, dendritic cells, and RB phosphorylation. We prioritized publications from 2020 to 2026 and retained earlier mechanistic studies and pivotal trials when they remained foundational. Evidence was grouped as clinical outcome, human translational, preclinical mechanistic, or registry evidence. This hierarchy prevents a blood immune correlate, a murine combination experiment, and an early clinical response from carrying the same inferential weight. Additional File 1 provides the full search record, selection boundaries, and update history.

Recent reviews have catalogued the dual immune effects of CDK4/6 inhibitors and summarized tumor-cell mechanisms, immune-cell mechanisms, and combination rationales (19–21). This review addresses a narrower translational problem: how an immune change can be judged usable rather than merely detectable. We make three distinctions that are not the organizing framework of those reviews. First, evidence is tiered as clinical outcome, human translational finding, preclinical mechanism, or untested proposal. Second, immune modulation is treated as a dynamic window rather than a stable drug-class property. Third, six sequencing models connect that window to partner-specific measurements and explicit reasons to stop or de-escalate.

The therapeutic window is the organizing unit. It begins when CDK4/6 inhibition produces a reproducible pharmacodynamic change and ends when adaptation, effector-cell constraint, or toxicity outweighs the benefit of further exposure. The evidence is organized around three questions: does treatment increase tumor visibility, does it preserve or improve effector-cell function, and can a partner exploit the resulting state without closing the safety window? **Table 1** separates clinical outcomes, human correlates, preclinical mechanisms, feasibility data, and proposed schedules. The distinction matters because the field has often moved from an immune signal to a combination strategy without showing that the signal persists, reaches the tumor, or changes patient outcome.

**Table 1:** Evidence labels and inference levels for CDK4/6 inhibitor immune-effect claims.

Evidence label	Claim supported	Examples in this review	Main boundary
Direct clinical outcome evidence	A defined regimen changes patient-level efficacy or toxicity	PACE and early CDK4/6 inhibitor plus ICI studies (16–18, 81, 82)	Does not demonstrate an immune mechanism unless paired immune readouts support it
Human translational evidence	On-treatment blood or tissue changes accompany CDK4/6 inhibitor plus endocrine therapy	Treg/MDSC and activated CD8+ T-cell changes (15)	Endocrine backbone, sampling compartment, and clinical context confound attribution
Preclinical mechanism evidence	CDK4/6 inhibition can alter a defined tumor or immune pathway under tested conditions	ERV/type III interferon, antigen presentation, T-cell activation, memory, PVR/PD-L1 regulation (8–15, 54)	Dose, duration, model, and drug-specificity limit clinical extrapolation
Combination feasibility evidence	A combination can be delivered or has preliminary activity in a selected setting	CDK4/6 inhibitor plus T-DM1 trials (93, 94) and retrospective reports summarized in reviews and timing perspectives (85–87)	Feasibility is not proof of a clinically usable immune state or an optimal sequence
Registry or trial-generating evidence	A trial is testing immune modulation or a proposed combination	NCT05766410 and NCT07198724	Registry status does not constitute efficacy evidence
Proposed therapeutic-window hypothesis	Biomarker-verified sequencing may improve net benefit	Models A-F and **Figures 2**, **3**	Requires prospective comparison with a concurrent or standard-care control

## 2 Breast cancer context and the conditional immune-priming framework

Breast cancer is commonly organized into HR+/HER2-negative, HER2-positive, and triple-negative breast cancer (TNBC) according to estrogen-receptor, progesterone-receptor, and HER2 expression. HR+/HER2-negative disease accounts for roughly 70% of cases, HER2-positive disease for 15% to 20%, and TNBC for about 15%, although distributions vary among populations (22). In early disease, local and systemic treatment depends on stage, subtype, and recurrence risk. In metastatic disease, endocrine therapy plus a CDK4/6 inhibitor is the usual first-line approach for most HR+/HER2-negative tumors. HER2-directed antibody combinations and ADCs anchor HER2-positive treatment. Chemotherapy remains central in TNBC, with pembrolizumab added in eligible PD-L1-positive metastatic disease and to neoadjuvant chemotherapy in high-risk early disease (1–6, 22–33).

These categories also have different natural histories. HR-positive disease often follows a longer course but carries a persistent risk of late recurrence. HER2-positive disease was historically aggressive, and HER2-directed therapy has substantially improved outcomes. TNBC has fewer established lineage targets, more early relapse, and a more aggressive metastatic course (22). Treatment itself reshapes the immune setting: endocrine therapy acts on hormone-responsive tumor and stromal compartments, HER2 antibodies recruit Fc receptor-dependent effector functions, chemotherapy and radiotherapy release antigens, and ADCs combine target-directed delivery with payload effects (28–35).

The immune contrast is real but not binary. TNBC is often more lymphocyte-rich than HR-positive breast cancer, although some TNBCs are immune excluded (36–41). Randomized trials have established ICI benefit in defined early and metastatic TNBC settings (23–27). Most HR-positive tumors are less inflamed, but a subset contains active immune niches. Immunopeptidomics adds a different layer. HR-positive and TNBC tumors share many source genes for MHC I peptides, whereas tumor-specific antigens are more frequent in TNBC (42). Low-dose palbociclib or abemaciclib can induce new HLA ligands in breast cancer cells, and abemaciclib-driven changes have been linked to RB-dependent chromatin remodeling (43, 44). Subtype therefore shapes both the baseline antigen landscape and the probability that a drug-induced change can be used.

The clinical and immune patterns create an apparent paradox. HR+/HER2-negative disease is the subtype in which CDK4/6 inhibitors have their largest established benefit, yet it is usually less T-cell-inflamed and less responsive to ICI than TNBC. A modest treatment-induced immune change may therefore be accessible in routine HR-positive therapy, but it begins from a less favorable immune state. Checkpoint response still depends on antigen availability, processing and presentation, dendritic-cell activation, T-cell expansion and trafficking, intratumoral access, effector function, and escape from suppression (45, 46). CDK4/6 inhibition can influence several steps, but none of the CDK4/6 inhibitor studies included in this review directly demonstrated control of the entire chain or *de novo* naïve-T-cell priming.

**Figure 1:**
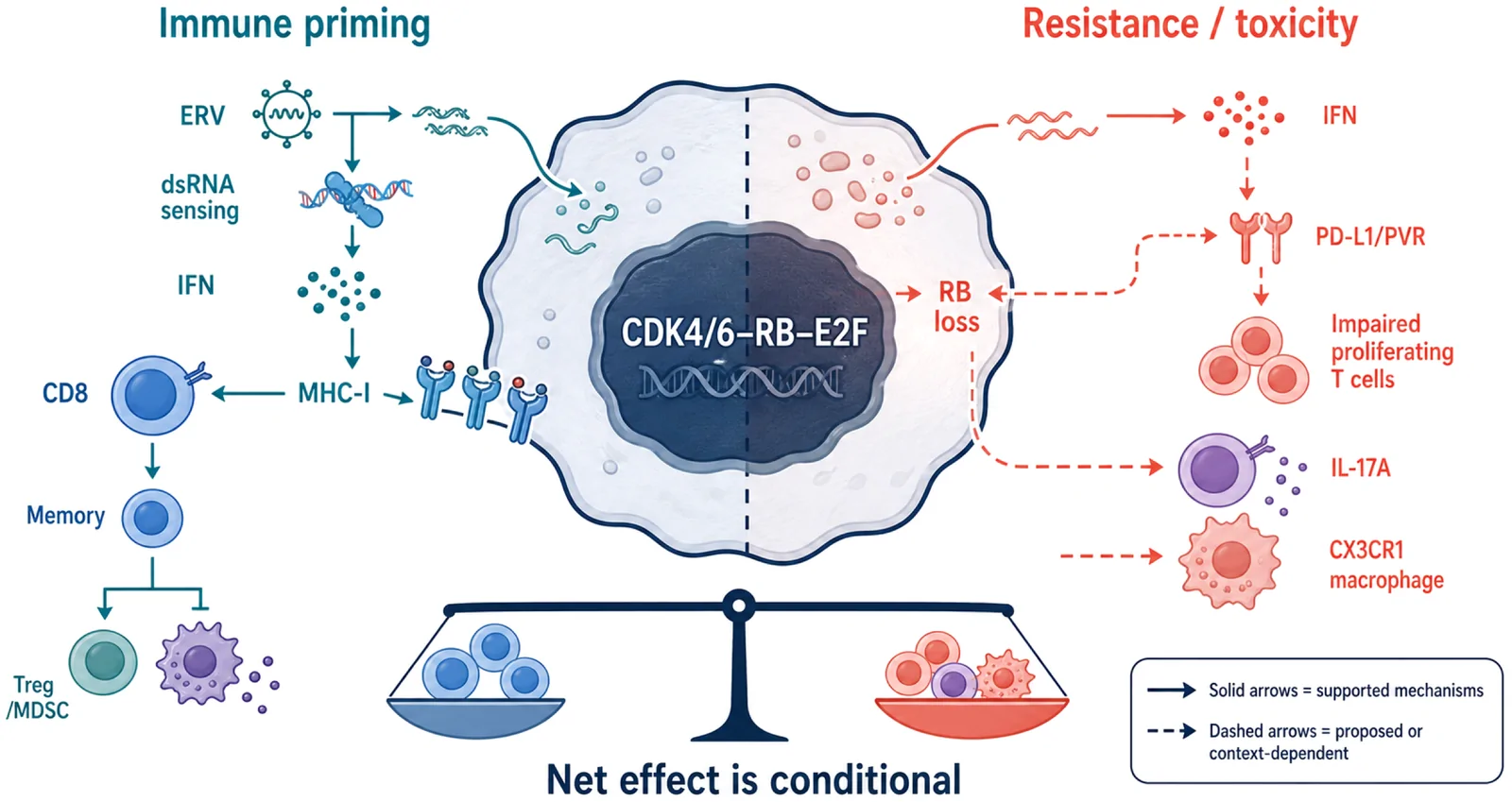
Conditional immune effects of CDK4/6 inhibitors. CDK4/6 inhibition can promote ERV-associated dsRNA sensing, interferon signaling, antigen presentation, CD8+ T-cell activation, memory formation, and reduction of suppressive Treg/MDSC compartments. The same treatment context can also be associated with chronic interferon-associated adaptation, context-dependent PD-L1/PVR regulation, effector-cell proliferative constraint, RB loss/E2F bypass, and IL-17A-CX3CR1+ macrophage-associated resistance. Solid arrows denote mechanisms directly supported by the cited literature, including ERV/interferon and antigen-presentation effects (8), T-cell activation and memory effects (9, 12–14), checkpoint-ligand and CD155/TIGIT regulation (11, 54, 55), and adverse myeloid remodeling (67, 71, 72). Dashed arrows denote context-dependent or proposed relationships. The balance depicts a conditional biological model, not a predicted clinical outcome.

The probability and meaning of a therapeutic window are therefore subtype dependent. HR-positive tumors may require a larger gain in tumor visibility or antigen-presenting-cell support; TNBC may begin with more T cells but still fail through immune exclusion or antigen-presentation loss; HER2-positive disease is conditioned by antibody-mediated Fc effector functions and prior HER2-directed therapy. The cancer-immunogram and immunoediting frameworks place tumor visibility, immune activation, trafficking, recognition, and suppression on the same path (45, 46). A CDK4/6-induced window can open through interferon signaling, antigen presentation, lower suppressive-cell burden, T-cell activation, or memory formation. It can close through cell-cycle bypass, immune exclusion, suppressive macrophages, marrow injury, hepatotoxicity, or pneumonitis. Tzetzo et al. provide a clinical example of this divergence: short progression-free survival was associated with cell-cycle and polo-like kinase signaling and macrophage accumulation, whereas the long-PFS group showed pretreatment immune signaling that intensified during therapy (47). **Figure 1** places these opposing trajectories within one model.

## 3 Tumor-intrinsic immune visibility

### 3.1 Endogenous retroviral signaling, nucleic acid sensing, and antigen presentation

Goel et al. linked cell-cycle control to tumor immune visibility. CDK4/6 inhibition activated endogenous retroviral elements, increased intracellular double-stranded RNA (dsRNA), induced type III interferon signaling, and enhanced antigen presentation (8). Tumor cells were therefore altered in two ways at once: proliferation slowed, and immune recognition increased.

CDK4/6-induced ERV reactivation primarily engages RNA sensing. The resulting dsRNA was associated with a viral-mimicry response, type III interferon production, and enhanced antigen presentation (8); the relative contributions of TLR3, RIG-I, and MDA5 were not resolved in that study. Type III interferon may be especially relevant in epithelial tumors because IFNLR1 expression is more restricted than the broadly distributed type I interferon receptor (8, 48). cGAS-STING belongs to a separate DNA-sensing route that is well established after radiotherapy (49, 50). A clinically relevant cGAS-STING response to CDK4/6 inhibition alone has not been demonstrated in breast cancer.

Recent work also supports a distinction between cytostatic and genotoxic forms of therapy-induced senescence. In breast cancer models, CDK4/6 inhibitor-induced senescence preserved antigen-presentation and T-cell-chemokine programs while producing less of the pro-tumorigenic, angiogenic SASP reported after DNA-damaging agents (51). This comparison does not establish an immune benefit in patients, but it strengthens the rationale for measuring both immune visibility and adverse secretory remodeling during a proposed conditioning interval.

Time changes the meaning of interferon signaling. Acute activation can increase antigen presentation and immune recruitment, whereas persistent signaling can drive PD-L1 expression, T-cell dysfunction, and adaptive resistance (52). Kong et al. found interferon, JAK/STAT, and checkpoint pathways enriched in tumors resistant to neoadjuvant palbociclib plus anastrozole (53). Interferon activation can therefore mark productive conditioning or an established resistant state. A single on-treatment measurement cannot distinguish the two without information on timing and cellular context.

### 3.2 RB-E2F control of immune-checkpoint ligands

The RB-E2F axis regulates checkpoint ligands at transcriptional and post-translational levels. Cyclin D-CDK4 controls PD-L1 protein stability through cullin 3-SPOP-mediated ubiquitination and proteasomal degradation (11). In TNBC models, Shrestha et al. found direct transcriptional regulation of both PVR (CD155) and CD274 by the pRB-E2F1 axis, with palbociclib generally suppressing both ligands under the tested conditions (54).

PD-L1 is therefore an unstable readout of CDK4/6 activity. Its direction of change depends on RB status, E2F activity, cell lineage, and treatment conditions, which favors serial measurement over a single baseline assay. PVR connects the same circuitry to the TIGIT-CD226 axis. Fassl et al. observed increased tumor-cell CD155 and lower TIGIT on tumor-infiltrating lymphocytes across breast cancer models, freshly resected tumors, and paired on-treatment biopsies (55). This pattern is compatible with greater CD155-CD226 signaling, but it differs from the earlier TNBC observations and does not yet identify patients for anti-TIGIT therapy.

### 3.3 RB dependence and unresolved immune consequences

RB competence is central to the canonical response to CDK4/6 inhibition, but current data do not establish RB as an immune gatekeeper. Tumors with RB loss or strong E2F bypass, including cyclin E-CDK2 activation or acquired RB1 mutations, are less likely to undergo durable G1 arrest (56–58). Pooled ctDNA analysis from the MONALEESA trials associated RB1 alterations with decreased ribociclib sensitivity (59). Pesch et al. showed that RB expression confers sensitivity to CDK4/6 inhibitor-mediated radiosensitization across breast cancer subtypes, but that study did not compare immune endpoints by RB state (60). RB activity is also more complex than a functional-versus-lost classification. Fourteen mono-phosphorylated RB isoforms have distinct interactomes and transcriptional outputs (61), and S249/T252 phosphorylation can connect RB to NF-κB and PD-L1 regulation in prostate models (62). These observations make RB abundance, function, and phosphorylation plausible modifiers of immune effects. Breast cancer studies have not yet demonstrated the proposed relationship, so RB is best treated as a mechanistic covariate rather than a stand-alone selector for immune-directed treatment.

## 4 Immune-cell-intrinsic effects

### 4.1 Regulatory T cells and suppressive myeloid compartments

CDK4/6 inhibitors influence immune cells directly. Goel et al. reported that CDK4/6 inhibition reduced Treg proliferation in preclinical models (8). Human translational evidence from Scirocchi et al. showed that patients with HR+, HER2-negative metastatic breast cancer treated with CDK4/6 inhibitors exhibited reductions in circulating Tregs, monocytic MDSCs, and polymorphonuclear MDSCs, along with increases in CD4+ T cells and CD137+CD8+ T cells, and a greater Treg reduction among responders (15). A separate analysis confirmed that the effector Treg subset (CD4+CD25+FOXP3highCD45RA−) is particularly reduced during CDK4/6 inhibitor therapy (15).

Peripheral immune monitoring has produced a broadly consistent signal, although the cohorts remain small. RIBECCA linked ribociclib treatment to adaptive-immune signature activation, lower immunosuppressive cytokine signaling, and a response-associated fall in circulating Tregs (63). A 28-day single-agent window study and a prospective 44-patient cohort also recorded changes in Tregs and selected myeloid or lymphocyte subsets (64, 65). These studies help define sampling times, but blood cannot establish what happened inside the tumor.

Paired-tissue studies tell a less uniformly favorable story. In CORALLEEN, ribociclib plus letrozole reduced antigen-presenting-cell and innate-immune signatures in Luminal B tumors relative to chemotherapy (66). Serial single-cell analysis then linked ribociclib resistance to suppressive cancer-to-myeloid communication, weaker IL-15/18-associated myeloid-to-CD8+ T-cell crosstalk, and diminished T-cell recruitment and activation (67). A fall in circulating suppressive cells cannot establish improved CD8+ access to tumor nests. Blood monitoring should therefore be paired with spatial tissue methods such as multiplex immunohistochemistry or imaging mass cytometry (39, 41).

NeoPAL reached a related conclusion from a different comparison. Letrozole plus palbociclib and chemotherapy produced broadly similar reductions in proliferation and immune-signature changes in high-risk luminal disease, yet cell composition and stromal remodeling varied across tumors (68). Together, NeoPAL and CORALLEEN show why one immune signature cannot serve as universal proof of a clinically usable immune state.

Most clinical immune-monitoring studies combined a CDK4/6 inhibitor with endocrine therapy. Estrogen-receptor signaling regulates immune and stromal compartments, and anti-estrogen treatment can change immune-cell composition, cytokines, and antigen-presentation states (34). Attribution therefore requires a fixed endocrine backbone, paired sampling, and, where feasible, an endocrine-only comparator. The contribution of each drug is likely to vary by endocrine agent, menopausal status, treatment line, and tumor subtype.

### 4.2 CD8+ T-cell activation, memory formation, and the CDK-dependence paradox

CDK4/6 inhibition changes T-cell function in ways that extend beyond Treg depletion. Palbociclib enhanced T-cell activation and cooperated with PD-1 blockade in preclinical models (9), while abemaciclib produced a T-cell-inflamed microenvironment and greater anti-PD-L1 activity (10). Other studies traced memory-associated CD8+ T-cell differentiation to an MXD4-MYC axis (12, 13). PD-1 blockade and CDK4/6 inhibition appear to augment different components of T-cell activation, which provides a mechanistic basis for sequencing rather than simple coadministration (14).

The memory data provide a rationale to test CDK4/6 inhibition before or around an immune-amplifying event. They do not establish that this sequence is superior to continuous concurrent treatment.

Antitumor immunity still requires lymphocyte proliferation, which creates a genuine scheduling problem for a cell-cycle inhibitor. Sensitivity to CDK4/6 inhibition varies with T-cell state and activation. The available data support preferential effects on Tregs and memory-associated CD8+ T-cell fate, while the effect on effector expansion depends on timing and exposure (8, 13, 15, 20). Heckler et al. showed that memory skewing was not a generic consequence of slower proliferation: inhibitors of CDK7, Aurora kinase, PLK1, or CDK1/2 did not reproduce the phenotype (13). In patient samples, palbociclib or abemaciclib increased the ratio of central-memory to effector precursors and reduced MYC target-gene scores in transitional CD8+ T cells (13).

Conventional oncology dosing may therefore be the wrong unit for an immune-conditioning study. Exposure should be mapped against antigen release, early T-cell activation, clonal expansion, effector function, memory formation, and toxicity recovery. The same dose can help during one phase and constrain the next.

Combination experiments reinforce the need for this phase-specific framing rather than a class-wide presumption of benefit. RANK pathway blockade restored CDK4/6 inhibitor sensitivity and enhanced T-cell infiltration in pRB-proficient TNBC models (69). Dual CDK4/6 and CDK7 inhibition enhanced immune-related signaling and antigen-specific T-cell responses in breast cancer models (70). Conversely, preclinical HR+/HER2-negative models suggest that CSF1R blockade may be needed to mitigate CDK4/6 inhibitor-associated macrophage-mediated immunosuppression (71). These studies nominate rational partners and measurable intermediates, not treatment-ready regimens.

CDK4/6-induced immune effects may also have a different dose-response curve from tumor-cell arrest. Published preclinical studies generally evaluated doses selected for substantial CDK4/6 pathway inhibition; direct dose- or schedule-comparison data for immune conditioning remain limited (19–21). The alternative is equally plausible: useful conditioning may require near-complete target engagement. Dose-de-escalation cohorts with paired immune pharmacodynamic endpoints can distinguish these possibilities.

### 4.3 *In vivo* evidence and model dependence

Animal studies provide direct evidence that CDK4/6-dependent immune changes can alter treatment response, but the models answer different questions. In immunocompetent mammary tumor models, palbociclib or abemaciclib increased T-cell activity and improved PD-1 or PD-L1 blockade (9, 10); related studies linked memory skewing, RANK or CDK7 cotargeting, macrophage-mediated resistance, and radiotherapy combinations to tumor control (12, 13, 69–73). Kumar et al. added an important counterexample: CDK4/6 inhibition reduced dendritic cells by acting on bone-marrow progenitors, and adoptive dendritic-cell transfer restored CD4-dependent tumor control during CDK4/6 inhibitor plus checkpoint blockade in mice (74). Xenografts establish tumor-cell radiosensitization but cannot reproduce an intact immune response (60, 75). These systems support causal mechanisms, yet their immune composition, hormone dependence, and dosing differ enough that no single model defines a clinical conditioning schedule.

### 4.4 Drug specificity of immune effects

Immune evidence is uneven across the three approved agents. Goel et al. demonstrated ERV activation and type III interferon induction with palbociclib and abemaciclib (8). T-cell activation was studied with palbociclib (9) and abemaciclib (10). Memory skewing through MXD4-MYC was shown with palbociclib and confirmed in samples from patients receiving palbociclib or abemaciclib, but has not been studied directly with ribociclib (12, 13). PD-L1 and PVR regulation were also examined primarily with palbociclib (11, 54). Human peripheral monitoring is most developed for palbociclib, with smaller datasets for ribociclib and abemaciclib (15). The registered phase 2 ORACLE-RIPA study (NCT05766410) compares immune modulation among palbociclib, ribociclib, and abemaciclib, each combined with letrozole, but the registry had not posted results when this review was updated. Agent-specific conditioning rules should wait for direct comparative data.

Recent syntheses likewise find tumor-cell and immune-cell effects in parallel, without resolving which agent, dose, or schedule is most useful (21) (**Table 2**). Treating the drugs as immunologically interchangeable obscures the question that comparative trials need to answer.

**Table 2:** Mechanistic evidence supporting conditional CDK4/6 inhibitor immune conditioning.

Mechanistic layer	Key evidence	Evidence level	Translational implication	Main limitation
ERV and interferon signaling	CDK4/6 inhibitor activates ERV elements, dsRNA sensing, type III interferon, and antigen-presentation programs (8)	Preclinical mechanism	A short exposure can be tested for pharmacodynamic immune visibility	Chronic interferon can support adaptive resistance (52)
Antigen presentation	MHC class I and antigen-processing programs rise in experimental models (8, 10)	Preclinical mechanism	Paired tissue can test whether the intended target engagement occurs	MHC class I loss may prevent effective recognition (107)
T-cell activation and memory	CDK4/6 inhibitor can enhance activation and bias newly activated CD8+ cells toward memory-like states (9, 12–14)	Preclinical mechanism with limited human support	Time exposure around activation and memory phases should be tested explicitly	Continuous exposure may constrain proliferative phases
Checkpoint-ligand dynamics	PD-L1 and PVR regulation is linked to cyclin D-CDK4 and RB-E2F circuitry (11, 54)	Preclinical mechanism	PD-L1/PVR should be sampled dynamically	Direction and magnitude are context-dependent
Suppressive-cell reduction	Circulating Tregs and MDSCs decline in selected patients receiving CDK4/6 inhibitor plus endocrine therapy (15)	Human translational evidence	Blood findings should be paired with spatial tissue analysis	Endocrine therapy and peripheral sampling confound attribution
Adverse immune remodeling	IL-17A-secreting gamma-delta T cells and CX3CR1+ macrophages can promote resistance (72)	Breast cancer preclinical mechanism	On-treatment monitoring may identify window closure	Prospective clinical validation is absent

## 5 Resistance and adverse immune remodeling

Resistance to CDK4/6 inhibitors is typically discussed through tumor-cell-intrinsic mechanisms: RB loss, cyclin E-CDK2 activation, E2F reactivation, PI3K pathway alterations, and endocrine resistance crosstalk (56–58). These mechanisms are relevant to immune conditioning because a tumor that bypasses CDK4/6 control may no longer show the same antigen-presentation or checkpoint-ligand changes. The direction of that effect has not been established by immune endpoints stratified by RB status. Baseline RB1 alterations were associated with reduced ribociclib sensitivity in the pooled MONALEESA analysis (59), whereas early ctDNA dynamics provided a separate response-monitoring signal in PALOMA-3 (76). Neither finding establishes RB1 or ctDNA as a selector for an immune sequence.

Resistance can also emerge through adverse immune remodeling. In HR+, HER2-negative breast cancer models, Petroni et al. showed that IL-17A-secreting gamma-delta T cells recruit and polarize CX3CR1+ macrophages, which suppress antitumor immunity and reduce CDK4/6 inhibitor activity (72). This pathway explains how treatment can reduce one suppressive compartment yet permit another to expand. The resulting state is an acquired immune escape program, not simply persistence of a pre-existing immune-cold tumor.

The same study linked CX3CR1+ macrophages to poor outcome in patients who received PD-1 blockade and radiotherapy, but it did not establish that radiotherapy reversed the IL-17A-driven program (72). Serial assessment of IL-17A, gamma-delta T cells, and macrophage state may help identify window closure. Whether radiotherapy or another partner can reverse this pathway in patients remains unknown.

## 6 Clinical translation with immune checkpoint inhibitors

### 6.1 Current ICI context in breast cancer

The role of ICIs is best established in TNBC. KEYNOTE-355 established pembrolizumab plus chemotherapy in PD-L1 CPS ≥10 metastatic TNBC (23). KEYNOTE-522 demonstrated event-free and overall survival benefit with pembrolizumab added to neoadjuvant chemotherapy in early-stage TNBC (24, 25). In HR+ disease, the evidence is maturing: KEYNOTE-756 and CheckMate 7FL showed that adding pembrolizumab or nivolumab to neoadjuvant chemotherapy improved pathologic complete response in high-risk ER+, HER2-negative early breast cancer (77, 78). I-SPY2 suggested pembrolizumab benefit in both HR+/HER2-negative and TNBC cohorts (79).

Selected HR+ tumors can respond to immunotherapy, particularly in high-risk early disease when ICIs are combined with chemotherapy. KEYNOTE-756 and CheckMate 7FL achieved their gains on a chemotherapy backbone that already provides antigen release and inflammatory signaling. CDK4/6 inhibition has not been shown to replace or augment that function. The problem also differs from TNBC, where baseline immune infiltration is generally higher and ICI benefit is established in defined settings (23–27, 38–40). Trials in HR+ disease must therefore test whether CDK4/6-induced conditioning expands the responsive fraction beyond chemotherapy-driven immune activation, rather than borrowing the TNBC treatment logic.

### 6.2 Monotherapy and clinical experience with immune combinations

CDK4/6 inhibitor monotherapy provides an efficacy reference without an endocrine or immune partner. In MONARCH 1, continuous abemaciclib at 200 mg twice daily produced an objective response rate of 19.7%, a clinical benefit rate of 42.4%, and median progression-free survival of 6.0 months in 132 heavily pretreated patients with HR+/HER2-negative metastatic breast cancer (80). The study did not include serial tumor immune endpoints. Most established clinical benefit in this subtype comes from endocrine combinations (1–6). Monotherapy therefore demonstrates tumor-intrinsic activity, not immune conditioning, while the ICI combinations below test whether immune amplification can add enough benefit to offset toxicity.

The most encouraging early signal came from the phase I/II study of palbociclib, pembrolizumab, and letrozole in HR+ metastatic breast cancer (Yuan et al.), which demonstrated feasibility and notable activity including complete responses in the front-line cohort (16). As a small, nonrandomized study, it remains hypothesis-generating rather than definitive.

The abemaciclib-plus-pembrolizumab experience was less favorable: a phase Ib study (Rugo et al.) showed antitumor activity but was associated with high rates of interstitial lung disease/pneumonitis and severe transaminase elevations, and the benefit-risk profile did not support further evaluation of that specific regimen (17). The ribociclib-plus-spartalizumab experience reinforced this pattern: limited efficacy and elevated hepatotoxicity, with hypertransaminasemia in 66.7% (AST) and 46.7% (ALT) of patients, including grade 3/4 events (18). On-target PD-1 occupancy and increased activated CD8+ T cells confirmed that immune engagement occurred, but the therapeutic window was closed by toxicity. CheckMate 7A8, testing nivolumab with palbociclib and anastrozole in primary ER+, HER2-negative breast cancer, also encountered substantial hepatotoxicity during safety evaluation (81). These studies show that some continuously coadministered CDK4/6 inhibitor plus ICI regimens can be limited by hepatotoxicity or pneumonitis; whether exposure separation improves safety remains untested.

The PACE trial added a caution from the post-CDK4/6 inhibitor setting: palbociclib plus fulvestrant did not improve progression-free survival compared with fulvestrant after prior CDK4/6 inhibitor and endocrine therapy, and the trial’s immune-containing arm was hypothesis-generating rather than supportive of late unselected immune intensification (82). Across published breast cancer trials, PD-1 or PD-L1 is the principal immune target tested clinically with a CDK4/6 inhibitor. PVR-TIGIT, macrophage-directed therapy, and dendritic-cell restoration remain preclinical strategies (54, 55, 71, 74).

### 6.3 Why continuous coadministration can close the window

Continuous coadministration assumes that conditioning and immune amplification should occur at the same time. The available data argue against that assumption. Many HR+ tumors begin with limited immune infiltration, and CDK4/6 inhibition alone may not supply enough antigen release or dendritic-cell activation (39, 40). Prolonged exposure can also constrain lymphocyte expansion even when it favors memory formation or reduces Tregs (8, 13, 15, 20). In the clinic, liver and lung toxicity has ended treatment before a durable immune benefit could develop (17, 18, 81). The basis of the hepatotoxicity remains uncertain and may include immune-mediated injury, pharmacokinetic interactions, and drug-specific off-target effects. A transient immune gain can therefore be lost through adaptation or toxicity during continued dosing.

A window-based design separates these phases. A brief CDK4/6 inhibitor run-in can be assessed for greater immune visibility, lower suppressive-cell burden, and preserved effector function before ICI exposure. The 9-gene immune signature received independent support in a small NeoPalAna cohort and illustrates a candidate integrated readout for prospective validation (83). **Table 3** shows why activity, immune engagement, and safety must be evaluated as separate outcomes.

**Table 3:** Clinical lessons from CDK4/6 inhibitor plus ICI studies.

Study	Regimen and setting	What the study establishes	Key safety boundary	What it does not establish
Yuan et al., 2021 (16)	Palbociclib, pembrolizumab, and letrozole; HR+ metastatic breast cancer	Feasibility and preliminary activity in a small nonrandomized cohort	Sample size limits efficacy inference	Superiority of any conditioning or concurrent schedule
Rugo et al., 2022 (17)	Abemaciclib plus pembrolizumab; HR+ metastatic breast cancer	Immune combination activity can be accompanied by substantial toxicity	ILD/pneumonitis and severe transaminase elevations	That exposure separation would improve safety or efficacy
Garrido-Castro et al., 2025 (18)	Ribociclib plus spartalizumab with or without fulvestrant	Immune engagement occurred	Grade 3/4 hepatotoxicity limited dosing	That activation translated into a usable therapeutic window
CheckMate 7A8 (81)	Nivolumab, palbociclib, and anastrozole; primary ER+/HER2-negative breast cancer	Safety experience in early disease	Substantial hepatotoxicity	Efficacy of the triplet or a preferred sequence
PACE (82)	Fulvestrant with palbociclib, with or without avelumab; post-CDK4/6 inhibitor HR+ metastatic breast cancer	Rechallenge does not broadly improve PFS in an unselected setting	Late intensification may add treatment without selection	That post-progression immune intensification is broadly useful
NCT05766410	Three CDK4/6 inhibitors plus letrozole; early ER+/HER2-negative disease	Comparative immune-modulation study design	Results pending public reporting	Any established drug-specific conditioning rule

Among the published combination studies identified in this review, no randomized trial has compared short-course conditioning with continuous concurrent treatment (16–18, 81, 82). The proposed sequence is supported by mechanism and by the toxicity of existing combinations.

## 7 Radiotherapy as an immune amplifier

### 7.1 Immunogenic radiotherapy biology

Radiotherapy is a compelling partner for CDK4/6 inhibitor-based immune conditioning because it can trigger tumor-cell death, antigen release, inflammatory cytokine signaling, and local microenvironmental remodeling. Radiation activates the cGAS-STING pathway: cytosolic DNA from damaged tumor cells is sensed by cGAS, which generates cGAMP, activating STING and downstream TBK1-IRF3-dependent type I interferon production (49). Radiation can also activate cellular RNA-sensing pathways, including MDA5 and downstream MAVS signaling, although this evidence is not specific to breast tumor cells (84). Vanpouille-Box et al. demonstrated that high single radiation doses induce the exonuclease TREX1, which degrades cytosolic DNA and attenuates STING-dependent immunogenicity, providing a biological explanation for why dose and fractionation affect immune outcomes (50). These findings make radiotherapy more than a local cytotoxic tool: it can serve as a programmable immune trigger, but only under schedules that preserve antigen release, innate sensing, and immune-cell participation.

### 7.2 CDK4/6 inhibitors and radiosensitization

Pesch et al. demonstrated that RB expression confers sensitivity to CDK4/6 inhibitor-mediated radiosensitization across breast cancer subtypes (60). This provides a mechanistic bridge between canonical CDK4/6-RB biology and radiotherapy combinations, although the reported endpoints were DNA repair and tumor response rather than immunity. Yang et al. showed that CDK4/6 inhibitors radiosensitize HR+/HER2-negative breast cancer cells through downregulation of DNA double-strand break repair and attenuation of NF-κB signaling, with synergy confirmed in orthotopic xenografts (75). An immunocompetent TNBC study tested abemaciclib at 50 mg/kg daily, focal radiotherapy at 8 Gy weekly for two doses, and anti-PD-L1 three times per week in 4T1 and EMT6 tumors. The triplet suppressed tumor growth and increased intratumoral CD4+ and CD8+ T cells, interferon-γ, and M1-like macrophages (73). These schedules provide a concrete preclinical starting point, not a clinically optimized regimen.

The STING pathway is positioned at the intersection of these mechanisms. Radiation activates STING, and CDK4/6 inhibition can enhance tumor-cell vulnerability to radiation-induced DNA damage. NF-κB signaling changed in one HR+, HER2-negative radiosensitization model (75), but its role as a shared integrating node in the combination remains to be tested. The specific contribution of STING to CDK4/6 inhibitor plus radiotherapy synergy has not been directly demonstrated.

### 7.3 Clinical regimens, safety, and resistance

Available clinical experience consists of small retrospective reports summarized in reviews and timing perspectives, with heterogeneous targets, drug-hold practices, and radiation schedules (85–87). Reported regimens include 8 Gy in one fraction, 20 Gy in five fractions, 30 Gy in ten fractions, and stereotactic courses ranging from 24 to 54 Gy in one to five fractions, most often for palliation. These reports have not revealed a consistent unexpected acute toxicity signal, but they lack standardized immune endpoints and cannot identify an immune-active fractionation schedule. Whether a particular radiation regimen can reverse IL-17A-driven macrophage-associated CDK4/6 inhibitor resistance remains unknown.

**Figure 2:**
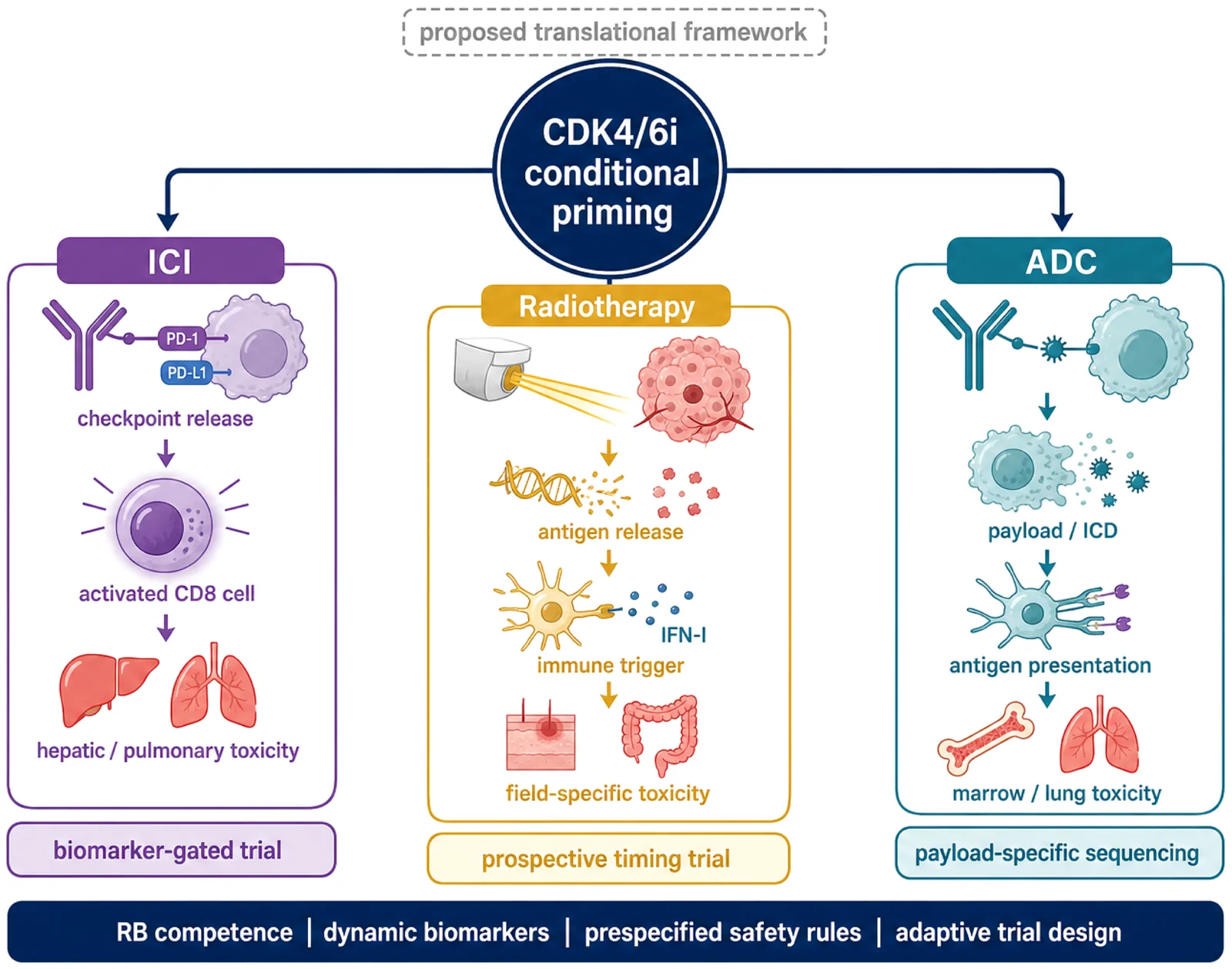
Combination strategy atlas for ICI, radiotherapy, and ADC partnerships. ICI, radiotherapy, and ADCs engage different potential routes to immune amplification. In this proposed framework, RB status and pathway activity are measured as candidate mechanistic modifiers rather than used as universal eligibility criteria; dynamic immune readouts and partner-specific safety assessments define the research questions. The figure does not present established treatment recommendations. Therapeutic vaccination and antigen-presenting-cell support are discussed separately in Section 9 because their evidence is currently preclinical.

A prospective sequence could use focal radiotherapy for antigen release and innate sensing, a protocol-defined CDK4/6 inhibitor exposure, and later ICI after documenting the intended immune change. RB status, lesion accessibility, marrow reserve, and prior resistance would be measured rather than assumed to define benefit. **Figure 2** places this hypothesis alongside ICI and ADC strategies, while **Table 4** lists partner-specific exploratory readouts.

**Table 4:** Partner modalities, evidence maturity, and questions for prospective testing.

Partner modality	Directly supported biology	Evidence maturity	Trial-generating question	Potential readouts and safety considerations
ICI	Checkpoint release can complement CDK4/6 inhibitor-associated immune changes (9, 10, 14)	Human combination data are early and mixed (16–18, 81, 82)	How does biomarker-gated sequencing compare with continuous concurrent exposure?	Paired tissue, CD8/Treg/MDSC dynamics, liver tests, pulmonary monitoring
Radiotherapy	Radiation provides antigen release and cGAS-STING-dependent innate sensing (49, 50); RB can condition radiosensitization (60, 73, 75)	Preclinical rationale with clinical reports summarized in reviews and timing perspectives (85–87)	Which radiotherapy dose, lesion, and CDK4/6 inhibitor schedule create a measurable immune state?	Lesion and marrow assessment, tissue interferon/MHC class I, field-specific toxicity, and CDK4/6 inhibitor hold interval as study variables
T-DXd and ADC sequencing	T-DXd has preclinical and ex vivo evidence of immunogenic cell-death-associated immune effects (35, 88)	Clinical CDK4/6 inhibitor plus ADC immune evidence is absent; T-DM1 feasibility exists (93, 94)	Can an ADC-specific antigen-release event be sequenced without reducing payload activity?	Agent-specific pharmacodynamics, marrow reserve, lung monitoring, immune-correlative endpoints
Endocrine therapy backbone	Endocrine therapy is standard in HR+ disease and can reshape immune contexture (34)	Clinical efficacy is established; immune-specific attribution is indirect	Which backbone permits interpretable CDK4/6 inhibitor immune pharmacodynamics?	Prespecified endocrine comparator, paired sampling, and attribution analysis
Therapeutic vaccination or dendritic-cell support	CDK4/6 inhibition can alter the HLA-ligand repertoire, while dendritic-cell loss can limit T-cell responses (42–44, 74, 97)	Immunopeptidomic and murine evidence; no clinical sequencing data	Can an induced HLA ligand be linked to cross-presentation and clonal T-cell expansion?	HLA-ligand identification, dendritic-cell abundance and function, TCR clonality, and vaccine-specific toxicity

## 8 Antibody-drug conjugates in the CDK4/6 inhibitor era

### 8.1 Why ADCs belong in the immune-conditioning discussion

ADCs now shape treatment across breast cancer subtypes. Trastuzumab deruxtecan (T-DXd) improves outcomes in HER2-positive, HER2-low, and HER2-ultralow disease (29–31), while sacituzumab govitecan is active in metastatic TNBC and heavily pretreated HR+ disease (32, 33). T-DXd also has immune effects in model systems. Its DXd payload induces immunogenic cell death through TLR4 and STING signaling in myeloid cells, increases antigen presentation to CD8+ T cells, and engages antibody-dependent cellular phagocytosis through the trastuzumab backbone (35). Viable human breast-cancer slices provide preliminary ex vivo support, with increases in HMGB1 release, tumor MHC class I, immune-cell CD86, granzyme B-positive cytotoxic T cells, and inflammatory cytokines after treatment (88). These findings are specific to T-DXd and do not establish a shared mechanism across topoisomerase I inhibitor ADCs. No published clinical immune-correlative study identified in this review has shown that adding a CDK4/6 inhibitor to an ADC produces a clinically usable immune state in patients.

### 8.2 ADC payload class and immune consequences

Payload class determines how this strategy should be tested. T-DXd has direct preclinical and ex vivo evidence of immunogenic cell death and myeloid activation (35, 88), but comparable immune-correlative data were not identified in this review for sacituzumab govitecan, datopotamab deruxtecan, or other topoisomerase I inhibitor ADCs (89–92). T-DM1 has a microtubule payload and different linker pharmacology (93, 94). The experimental question is agent specific: can ADC-induced antigen release be separated from CDK4/6 inhibition without reducing payload cytotoxicity or effector-cell function? **Table 4** treats ADC sequencing accordingly.

### 8.3 Direct clinical evidence and limitations

Clinical CDK4/6 inhibitor-ADC studies have addressed feasibility rather than immune mechanism. Ribociclib plus T-DM1 and palbociclib plus T-DM1 were tested in HER2-positive advanced breast cancer (93, 94), but neither study established immune modulation as a mediator of benefit. ERADICATE (NCT07198724) is recruiting patients with CDK4/6 inhibitor-resistant HR+/HER2-low or HER2-ultralow metastatic disease for elacestrant plus T-DXd; its primary endpoints are dose and efficacy, not a clinical immune mechanism. Target expression, internalization, linker and payload class, drug efflux, and the tumor microenvironment will continue to shape ADC response (89). T-DXd-associated interstitial lung disease is an additional constraint when an immune or radiation partner is considered (95).

The sequence can be studied in three settings: an ADC after CDK4/6 inhibitor resistance, concurrent ADC and CDK4/6 inhibition in a biologically selected tumor, or ADC-induced antigen release followed by CDK4/6-directed immune stabilization (90, 91). The last setting fits the conditioning hypothesis most closely and has the least clinical evidence (92). Biomarker selection will matter. In preclinical HER2-positive systems, p95HER2 drove an immunosuppressive program and reduced T-DXd efficacy (96), illustrating how target biology can override a plausible immune sequence.

## 9 Therapeutic vaccination and antigen-presenting-cell support

CDK4/6 inhibition may create vaccine-relevant antigenic substrates. Quantitative immunopeptidomics first showed that palbociclib and abemaciclib alter the MHC I repertoire in melanoma cells (97). In breast cancer cell lines, low-dose palbociclib or abemaciclib induced hundreds of HLA ligands, including peptides from G1/S pathway proteins (43). Analysis of 26 primary breast tumors found substantial overlap in peptide source genes between HR-positive disease and TNBC, but tumor-specific antigens were more frequent in TNBC and associated with leukocyte infiltration and survival (42). More recently, abemaciclib reprogrammed the immunopeptidome in HR-positive and TNBC models through RB-dependent chromatin and transcriptomic changes (44). These studies show changes in peptide display. They do not establish *de novo* naïve-T-cell priming in patients.

This boundary places antigen-presenting-cell function at the center of the combination rationale. Kumar et al. found that CDK4/6 inhibition depleted dendritic cells through effects on bone-marrow progenitors; restoring dendritic cells during CDK4/6 inhibition and checkpoint blockade increased tumor control and CD4+ T-cell responses in mice (74). Peptide, neoantigen, mRNA, or dendritic-cell vaccination is therefore a testable partner when treatment creates recognizable HLA ligands but antigen-presenting-cell function is limiting. A mechanistic study could select an HLA ligand induced by the chosen inhibitor, document cross-presentation and clonal expansion, and compare vaccine or dendritic-cell support with checkpoint blockade alone. No clinical study identified in this review has shown that this sequence improves breast cancer outcomes.

## 10 Candidate biomarkers for testing the therapeutic window

### 10.1 Tumor-cell-cycle biomarkers

RB status is the most obvious cell-cycle biomarker, but it is insufficient alone. CDK4/6 inhibitor sensitivity is shaped by cyclin E-CDK2 activation, E2F output, p16 status, PI3K pathway alterations, and acquired resistance mechanisms (56–59). Tzetzo et al. recently showed that high baseline cyclin A or E protein levels correlate with shorter progression-free survival on CDK4/6 inhibitors, and that cyclin E-high tumors show an inverse correlation with CD8+ T-cell detection, suggesting that cell-cycle and immune features are coupled at baseline (47). Functional studies also support cyclin D1/CDK4-mediated resistance that can be reversed experimentally by PI3K/mTOR inhibition (98).

Serial blood-based profiling can complement tissue measurements but should be treated as a resistance and response-monitoring layer, not a surrogate for the microenvironment. In a 51-patient prospective cohort, elevated blood tumor mutational burden or copy-number burden predicted poorer outcome on endocrine therapy plus CDK4/6 inhibition, and rising copy-number burden preceded imaging progression in a subset (99). A real-world ctDNA analysis independently found enrichment of ESR1 and RB1 alterations after CDK4/6 inhibitor exposure (100). CTC transcriptional profiling has identified candidate baseline and progression signatures, while serial single-cell genomics of neoadjuvant specimens has shown convergent subclonal evolution during endocrine plus CDK4/6 therapy (101, 102). These observations motivate biomarker-stratified trials, but their assay thresholds and prospective utility remain unresolved. A recent integrated biomarker landscape and a liquid-biopsy review both underscore this gap (103, 104).

### 10.2 Immune contexture

TILs remain one of the most established immune biomarkers in breast cancer (36–38). For CDK4/6 inhibitor conditioning, baseline TILs should be interpreted together with CD8+ T-cell localization (intratumoral *vs*. stromal *vs*. excluded), T-cell exhaustion markers, Treg abundance, MDSC levels, macrophage polarization (M1/M2 ratio, CX3CR1 status), and antigen-presentation machinery integrity. Bassez et al. demonstrated through single-cell profiling that anti-PD-1 response in breast cancer involves coordinated changes across tumor, immune, and stromal compartments (105). Spatial methods are essential because immune-cell abundance does not reveal whether lymphocytes enter tumor nests, and spatially excluded phenotypes have been associated with anti-PD-1 resistance in TNBC (39, 41).

### 10.3 Interferon and antigen-presentation signatures

ERV activation and interferon signaling make interferon-response genes such as MX1, OAS1, and IFIT1, MHC class I expression, and antigen-processing components including TAP1/2 and beta-2 microglobulin candidate pharmacodynamic markers. Immunopeptidomics adds a functional layer by showing which HLA ligands are actually displayed and whether a drug-induced peptide could support an antigen-directed strategy (42–44, 97). These signatures must be interpreted dynamically. Acute interferon induction after CDK4/6 inhibitor exposure may accompany productive immune conditioning, whereas chronic signaling can accompany dysfunction and adaptive resistance (52, 53). The NeoPalAna endocrine-resistant cohort showed upregulation of interferon, JAK/STAT, and immune-checkpoint pathways in resistant tumors, illustrating that pathway activation can accompany resistance as well as sensitivity (53).

### 10.4 Checkpoint-ligand and alternative checkpoint dynamics

PD-L1 should be measured but not overinterpreted, given context-dependent regulation (11, 54). PVR should be incorporated because it connects CDK4/6-RB-E2F signaling to the TIGIT immune axis (54, 55). A dynamic panel could include PD-L1, PVR, TIGIT, LAG-3, TIM-3, and CD73, but selection should be guided by changes after CDK4/6 inhibitor exposure rather than a generic immune panel. The 9-gene immune signature reported by Felip et al., encompassing Treg, interferon-γ, PD-1, and TIS (tumor inflammation signature), is an example of a composite biomarker that captures multiple immune dimensions and warrants validation in prospective CDK4/6 inhibitor conditioning studies (83). Neither the KIMA signature nor any individual checkpoint or interferon measure should yet be used as a selection test outside prospective validation.

### 10.5 Safety biomarkers

The therapeutic window is clinical as well as biological. Liver-function reserve is critical for ribociclib and ICI combinations. Lung history and prior pneumonitis risk are critical for abemaciclib, ICIs, radiotherapy, and T-DXd. Marrow reserve is critical for radiotherapy, ADCs, and all CDK4/6 inhibitors. Autoimmune history matters for ICI candidacy. These organ-specific constraints are documented across the relevant combination studies and safety syntheses (17, 18, 81, 85, 87, 89, 93–95) and should be incorporated into trial eligibility criteria, dose-hold rules, and stopping boundaries.

### 10.6 A candidate core panel for exploratory studies

One feasible core panel for exploratory studies would cover five domains: RB1 status by immunohistochemistry or genomic profiling as a measure of cell-cycle competence (56–59); paired HLA-A/B/C and antigen-processing measurements (8, 42–44); standardized stromal TIL assessment (36, 37), supplemented by spatial CD8+ T-cell density where validated; a composite immune gene signature (83); and serial ALT, AST, and bilirubin when ICI escalation is studied. This panel is proposed to test the framework. Its feasibility, assay concordance, and decision thresholds have not been established across centers.

### 10.7 A proposed decision hierarchy for prospective evaluation

No validated biomarker threshold for escalation after CDK4/6 inhibitor conditioning was identified in the studies and biomarker syntheses reviewed here (83, 103, 104). As a hypothesis-generating design, we separate eligibility, escalation, and continuation. Eligibility would assess a responsive cell-cycle axis and organ reserve. Escalation would require paired evidence that antigen presentation is preserved and that a tumor effector signal is interpretable. Continuation would depend on persistence of that signal without prohibitive liver, lung, marrow, or radiation-field toxicity. Prospective work must establish within-patient change thresholds, assay reproducibility, and handling of discordant blood and tissue results. **Table 5** illustrates this proposed hierarchy for each partner modality.

**Table 5:** Illustrative modality-specific hypotheses for the conditional immune window.

Partner modality	Proposed biological trigger	Candidate condition to evaluate	Research comparison	Potential stopping signal	Evidence boundary
ICI	Short CDK4/6 inhibitor exposure to alter immune visibility or suppressive-cell balance	Preserved antigen presentation, an interpretable effector signal, and adequate organ reserve	Compare biomarker-gated ICI introduction with concurrent or standard-care control	Absent pharmacodynamic engagement or treatment-limiting liver, lung, or marrow toxicity	Clinical combination data are mixed; sequence superiority is unproven (16–18, 81, 82)
Radiotherapy	Lesion-directed antigen release and radiation-triggered innate sensing	RB status and pathway activity as candidate modifiers, together with target-lesion feasibility, marrow reserve, and an immune-relevant post-radiation signal	Compare prespecified CDK4/6 inhibitor timing with concurrent exposure; evaluate subsequent ICI in a separately defined study cohort	Field-specific toxicity, absent immune visibility, or adverse myeloid remodeling	Mechanistic rationale is strong; prospective schedule-defining evidence is limited (49, 50, 60, 73, 75, 85–87)
ADC	Payload-specific tumor-cell death and antigen release	Target expression, payload-compatible timing, organ reserve, and a documented immune correlate when feasible	Compare separated and concurrent ADC/CDK4/6 inhibitor exposure while testing immune stabilization as a mechanistic endpoint	Payload-limiting toxicity, loss of target or payload sensitivity, or absent immune engagement	T-DXd immune data are preclinical or ex vivo; published clinical studies identified in this review do not establish a clinically usable immune state with CDK4/6 inhibitor plus ADC treatment (35, 88, 89, 92–96)
Therapeutic vaccination or dendritic-cell support	Treatment-induced HLA ligands or reduced dendritic-cell availability	Reproducible HLA-ligand induction, competent antigen presentation, and a measurable vaccine-specific or CD4+ T-cell response	Compare vaccine or dendritic-cell support with checkpoint blockade or conditioning alone	No cross-presentation or clonal response, or treatment-limiting immune toxicity	The rationale is preclinical; clinical benefit in breast cancer is untested (42–44, 74, 97)

## 11 Hypothesis-generating sequencing models

**Figure 3:**
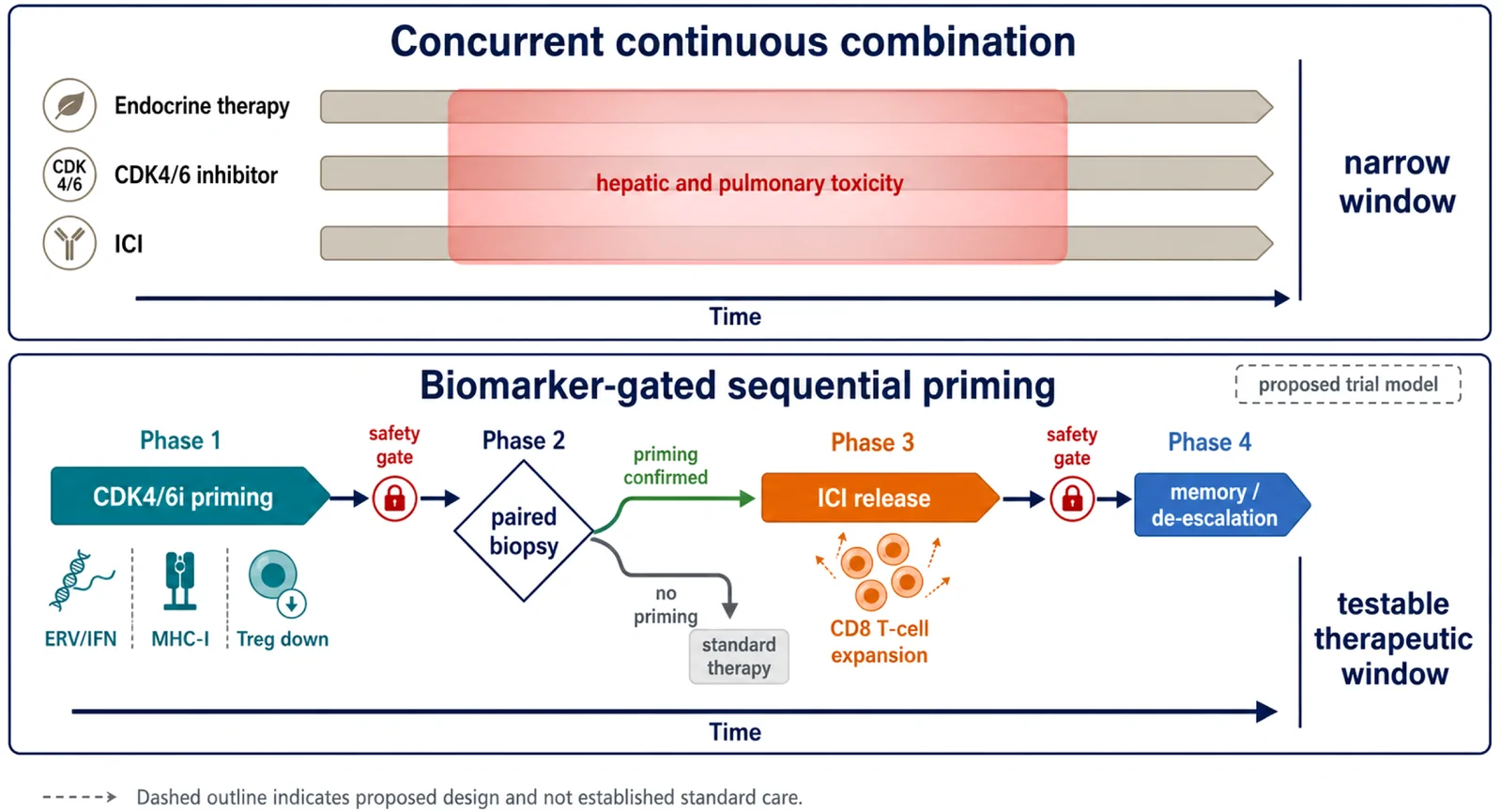
Therapeutic-window model for CDK4/6 inhibitor immune conditioning. The upper panel depicts the toxicity overlap reported with continuous concurrent endocrine therapy, CDK4/6 inhibition, and ICI treatment (17, 18, 81). The lower panel presents a proposed trial design in which a CDK4/6 inhibitor run-in is followed by paired biopsy assessment and biomarker-gated escalation. The sequential pathway is a testable design, not established standard care.

The following six sequencing models are an original, hypothesis-generating framework derived from the evidence reviewed above. They are not synthesized treatment recommendations. Each identifies a biological trigger, a timing problem, and a measurable reason to continue, pause, or stop treatment. **Figure 3** contrasts continuous treatment with biomarker-gated escalation, and **Figure 2** maps partner-specific routes.

Model A. Short-course CDK4/6 inhibitor conditioning followed by ICI. HR-positive tumors with partial immune competence but limited baseline inflammation provide one setting in which to test this design. A run-in of empirically determined duration would measure antigen presentation, suppressive-cell burden, and T-cell state. An ICI could then be introduced only in a research setting with a prespecified pharmacodynamic signal, preferably from paired tumor tissue rather than blood alone.

Model B. Radiotherapy-triggered activation with CDK4/6 modulation and later ICI. Radiotherapy supplies antigen release and innate sensing (49, 50, 84). Exploratory cohorts could vary the lesion, dose, fractionation, and CDK4/6 inhibitor hold interval. RB function, lesion accessibility, marrow reserve, and the post-radiation immune signal would be measured as candidate modifiers rather than used as established selection rules.

Model C. Cytotoxic therapy followed by cell-cycle-directed stabilization. PREDIX LumB randomized paclitaxel before or after palbociclib plus endocrine therapy (106). Although not an immune-conditioning trial, it shows that sequence can be tested and that a proliferation-dependent partner should not be treated as interchangeable across concurrent and sequential schedules.

Model D. ADC-induced antigen release followed by immune stabilization. The ADC provides the initial tumor-cell death event. CDK4/6 inhibition follows after early payload activity, with immune correlates used to test preservation of antigen presentation or memory-associated T-cell states. A theoretical risk is loss of payload activity when tumor proliferation is suppressed too early; existing CDK4/6 inhibitor-ADC studies establish feasibility but do not resolve this scheduling question (89–95).

Model E. Endocrine plus CDK4/6 inhibitor backbone with selective immune intensification. In HR+, HER2-negative disease, immune treatment can be restricted to patients who develop favorable changes during standard endocrine plus CDK4/6 therapy. A fixed endocrine backbone is essential because endocrine therapy itself alters the immune contexture (34).

Model F. De-escalation after window closure. Suppressive macrophage states, persistent interferon signaling, T-cell dysfunction, IL-17A-driven remodeling, or unacceptable toxicity are candidate signs of window closure (52, 53, 67, 71, 72). Prospective studies could test whether a planned treatment hold, de-escalation, or modality switch is safer and more informative than continuing an unproductive exposure.

## 12 Research design considerations

These proposals treat the immune window, rather than the maximum tolerated combination dose alone, as the design unit. A study based on this framework would define the biological change of interest, its expected timing, the measurement method, the condition for partner introduction, and a toxicity boundary. Paired tissue and blood at baseline, after conditioning, and during combination treatment could help a negative trial locate the failed step: target engagement, immune visibility, effector preservation, microenvironmental compatibility, or clinical amplification (**Table 6**).

**Table 6:** Illustrative measurements for testing a therapeutic window.

Analytical domain	Supporting evidence	Candidate measurement	Interpretive concern	Question for prospective testing
Cell-cycle competence	RB dependence and resistance biology (56–59)	RB1 status, E2F output, cyclin E/CDK2 features, ctDNA	RB loss or E2F bypass	Does pathway activity identify tumors capable of responding to CDK4/6 inhibition?
Immune visibility	ERV, interferon, and antigen-presentation studies (8, 10)	MHC class I, β2M, TAP1/2, interferon-response genes	No antigen-presentation increase	Should partner introduction depend on a demonstrated increase in immune visibility rather than dose delivery?
Effector preservation	T-cell activation and memory studies (9, 12–14)	CD8 state, TCR clonality, proliferation/exhaustion markers	Effector suppression or dysfunctional expansion	How do exposure duration and partner timing affect effector preservation?
Microenvironmental compatibility	Treg/MDSC reduction and macrophage resistance studies (15, 72)	Spatial CD8 localization, Tregs, MDSCs, macrophage state	Immune exclusion or adverse myeloid remodeling	Which partner best addresses the observed resistance state?
Clinical safety reserve	ICI, radiotherapy, ADC, and CDK4/6 inhibitor combination experience (17, 18, 81, 85, 87, 93, 94)	Liver tests, pulmonary assessment, CBC, radiotherapy field history	Toxicity closes the window before benefit	Can prespecified safety signals identify window closure before irreversible toxicity?
Clinical amplification	Combination studies and future adaptive trials	Objective endpoint plus validated pharmacodynamic correlate	Immune change does not translate into patient benefit	Does the proposed sequence improve an objective endpoint relative to concurrent or standard care?

Candidate exploratory endpoints include interferon-response gene induction, MHC class I changes, treatment-induced HLA ligands, CD8+ T-cell activation, Treg and MDSC dynamics, macrophage-state shifts, T-cell receptor clonality, PD-L1 and PVR changes, ctDNA dynamics, and spatial immune infiltration. A study would select a limited set tied to its proposed mechanism. Partner-specific safety monitoring would cover liver injury, pneumonitis, marrow suppression, and radiation-field toxicity.

Adaptive designs offer one way to study this question. After a short run-in and early immune assessment, research participants could enter an ICI, radiotherapy, ADC, vaccine or dendritic-cell, or endocrine-continuation cohort according to prespecified study rules. Early-disease window studies permit paired tissue collection before surgery but require narrow safety boundaries. Metastatic studies allow more flexible serial blood sampling and lesion-directed interventions. ORACLE-RIPA (NCT05766410) is designed to compare immune modulation by palbociclib, ribociclib, and abemaciclib, although no results were posted in the registry when this review was updated.

## 13 Limitations

The evidence thins as it moves from mechanism to schedule. Most mechanistic studies are preclinical; human translational cohorts are small and often rely on peripheral blood or exploratory tissue endpoints. ICI combinations are heterogeneous and have drug-specific liver and lung toxicities. Radiotherapy studies rarely include prospective immune endpoints, ADC-CDK4/6 combinations have not established an immune mechanism in patients, and vaccine or dendritic-cell strategies remain preclinical in this setting (15–18, 35, 63–68, 74, 81, 85–88, 93, 94). No comparative trial has tested short-course conditioning against continuous concurrent treatment. No breast cancer study has linked an immune outcome to a defined RB phosphorylation state.

Subtype is an incomplete proxy for immune state. Some HR+ tumors are inflamed, some TNBCs are immune excluded, and immune and stromal niches vary within and across conventional breast cancer subtypes (39–41). Patient selection will require dynamic biomarkers in addition to histologic subtype.

The six sequencing models are author-proposed research frameworks, not treatment algorithms. Their value depends on showing that the proposed immune window was created, used by the partner therapy, and closed before toxicity or resistant remodeling dominated.

## 14 Conclusions and research priorities

CDK4/6 inhibitors alter tumor visibility and immune-cell state across experimental systems. ERV reactivation, interferon signaling, antigen presentation, RB-E2F-dependent checkpoint regulation, Treg reduction, and MXD4-MYC-associated memory formation can favor antitumor immunity. The same treatment can also constrain effector expansion or select IL-17A-CX3CR1-positive macrophage programs that support resistance.

Used in the operational sense defined here, conditional immune priming captures this tension more accurately than classifying CDK4/6 inhibitors as uniformly immune activating or immune suppressive. It changes the development question: when does a drug create a usable immune state, which partner can exploit it, and when should exposure stop? Five priorities follow:

1. Window-of-opportunity trials need to establish the duration of CDK4/6 exposure that produces reproducible changes in interferon signaling, MHC class I, treatment-induced HLA ligands, T-cell activation, and suppressive-cell compartments.
2. Direct comparisons should determine which CDK4/6 inhibitor, dose, and schedule provide the most favorable immune profile. Results from ORACLE-RIPA would be informative when publicly reported.
3. A composite biomarker should combine interferon-response genes, antigen-presentation markers, spatial CD8+ features, Treg and MDSC dynamics, and ctDNA, building on emerging signatures (47, 68).
4. Randomized early-phase trials should compare biomarker-gated sequencing with concurrent treatment for ICI, radiotherapy, ADC, and antigen-presenting-cell-directed partners.
5. Protocols should prespecify hold, de-escalation, and modality-switch rules when biomarkers indicate window closure or adverse remodeling.

The next step is empirical window validation. Timing, mechanism, and toxicity must be measured together; otherwise, another active combination may still fail because the useful immune state was never created or was allowed to disappear.

## Data Availability

The datasets presented in this study can be found in online repositories. The names of the repository/repositories and accession number(s) can be found in the article/**Supplementary Material**.
