# Supplementary material for: CDK4/6 inhibitors as conditional immune-priming agents in breast cancer: therapeutic windows, sequencing strategies, and rational combinations with immunotherapy, radiotherapy, and antibody-drug conjugates
Source: Front Immunol. 2026 Aug 19;17:1934879. doi: 10.3389/fimmu.2026.1934879 (PMC13533973; doi:10.3389/fimmu.2026.1934879)
Supplement: Supplementary file 1 [file Table1.docx]

**Additional File 1. Narrative Search Record and Evidence-Selection Boundaries**

# **Purpose**

This supplement documents the reproducible search framework used for the narrative review, *CDK4/6 inhibitors as conditional immune-priming agents in breast cancer: therapeutic windows, sequencing strategies, and rational combinations with immunotherapy, radiotherapy, and antibody-drug conjugates*. It is not a systematic-review protocol and does not claim PRISMA-compliant study selection.

# **Sources and timing**

Targeted searches and metadata checks were performed in PubMed, Web of Science, Crossref, and ClinicalTrials.gov between October 2024 and May 2026. Reference metadata and DOI fields were rechecked in July 2026. Reviewer-directed searches for immunopeptidomics, therapeutic vaccination, dendritic cells, RB phosphorylation, in vivo models, and radiotherapy schedules were also conducted in July 2026. The searches prioritized peer-reviewed studies published from 2020 onward, while retaining older seminal mechanistic studies, pivotal efficacy trials, and landmark radiobiology studies where a recent source could not replace the original evidence.

# **Core search concepts**

Searches combined breast-cancer and CDK4/6 inhibitor terms with one or more of the following concept blocks:

| **Topic** | **Representative terms** |
| --- | --- |
| Disease and intervention | breast cancer OR breast neoplasm; CDK4/6 inhibitor OR palbociclib OR ribociclib OR abemaciclib |
| Tumor immune biology | immune microenvironment OR antigen presentation OR immunopeptidome OR HLA ligand OR endogenous retrovirus OR viral mimicry OR interferon OR PD-L1 OR PVR OR CD155 OR TIGIT |
| Immune cells and resistance | regulatory T cell OR Treg OR MDSC OR macrophage OR dendritic cell OR therapeutic vaccination OR neoantigen vaccine OR gamma-delta T cell OR T-cell memory OR immune resistance |
| Immunotherapy | immune checkpoint inhibitor OR PD-1 OR PD-L1 OR pembrolizumab OR nivolumab OR avelumab OR spartalizumab |
| Radiotherapy | radiotherapy OR radiation OR radiosensitization OR fractionation OR cGAS-STING OR DNA repair OR IL-17A OR macrophage resistance |
| ADCs | antibody-drug conjugate OR trastuzumab deruxtecan OR T-DXd OR trastuzumab emtansine OR T-DM1 OR sacituzumab govitecan |
| Biomarkers and monitoring | RB1 OR RB phosphorylation OR E2F OR cyclin E OR ctDNA OR circulating tumor cell OR transcriptomic signature OR spatial profiling |

# **Selection boundaries**

Studies were retained when they directly addressed at least one of the following questions: (1) tumor-intrinsic immune visibility after CDK4/6 inhibition; (2) immune-cell-intrinsic effects or adverse immune remodeling; (3) clinical safety or efficacy of CDK4/6 inhibitor monotherapy or combinations; (4) radiotherapy or ADC partner biology relevant to sequencing; (5) biomarkers that could define an immune window; (6) therapeutic vaccination or antigen-presenting-cell support; or (7) in vivo evidence and radiotherapy regimen details relevant to resistance.

Clinical trial reports, translational cohorts, mechanistic studies, and trial registry records were assigned separate evidence labels. Conference abstracts and registry records were used only for the limited claims they could support and were not treated as proof of clinical benefit or mechanism. Studies were excluded when they were unrelated to CDK4/6 inhibition, did not address breast cancer or a directly transferable mechanistic question, or lacked relevance to the review's therapeutic-window framework.

# **Evidence handling**

The review distinguishes direct clinical outcome evidence, human translational evidence, preclinical mechanistic evidence, combination feasibility evidence, registry or trial-generating evidence, and proposed therapeutic-window hypotheses. The evidence label is intended to prevent direct clinical claims from being inferred from immune correlates, animal models, or trial registration alone.

Generative AI was used for language refinement, literature organization, and schematic-figure drafting, but not as an evidence source or for study-eligibility decisions. Authors reviewed all citations, metadata, figures, and interpretive claims before finalization.
